# Systematic Evaluation and Comparison of Statistical Tests for Publication Bias

**DOI:** 10.2188/jea.15.235

**Published:** 2005-11-07

**Authors:** Yasuaki Hayashino, Yoshinori Noguchi, Tsuguya Fukui

**Affiliations:** 1 Department of Epidemiology and Healthcare Research, Kyoto University Graduate School of Medicine.; 2 Division of General Internal Medicine, Department of Medicine, Fujita Health University School of Medicine.; 3 St Luke’s International Hospital.

**Keywords:** Meta-Analysis, Cochrane library, funnel plots, statistical tests, Publication Bias

## Abstract

BACKGROUND: This study evaluates the statistical and discriminatory powers of three statistical test methods (Begg’s, Egger’s, and Macaskill’s) to detect publication bias in meta-analyses.

METHODS: The data sources were 130 reviews from the Cochrane Database of Systematic Reviews 2002 issue, which considered a binary endpoint and contained 10 or more individual studies. Funnel plots with observers’ agreements were selected as a reference standard. We evaluated a trade-off between sensitivity and specificity by varying cut-off p-values, power of statistical tests given fixed false positive rates, and area under the receiver operating characteristic curve.

RESULTS: In 36 reviews, 733 original studies evaluated 2,874,006 subjects. The number of trials included in each ranged from 10 to 70 (median 14.5). Given that the false positive rate was 0.1, the sensitivity of Egger’s method was 0.93, and was larger than that of Begg’s method (0.86) and Macaskill’s method (0.43). The sensitivities of three statistical tests increased as the cut-off p-values increased without a substantial decrement of specificities. The area under the ROC curve of Egger’s method was 0.955 (95% confidence interval, 0.889-1.000) and was not different from that of Begg’s method (area=0.913, p=0.2302), but it was larger than that of Macaskill’s method (area=0.719, p=0.0116).

CONCLUSION: Egger’s linear regression method and Begg’s method had stronger statistical and discriminatory powers than Macaskill’s method for detecting publication bias given the same type I error level. The power of these methods could be improved by increasing the cut-off p-value without a substantial increment of false positive rate.

Meta-analysis is increasingly used to summarize health-related evidence as an aid to clinical decision making. In meta-analysis, however, a kind of selection bias may affect the validity of conclusions, because studies with statistically significant results are more likely to be submitted and published or published more rapidly than those with non-significant results.^1^ Uncritically combining only the identified published studies may lead to an incorrect conclusion. Meta-analyses should therefore be scrutinized for the possible presence of such biases.^2^ A prospective registration of trials is encouraged through trial registries,^3^ but publication bias still remains a serious problem.

Because of their simplicity, funnel plots, the graph of estimates of the effect of each trial versus sample size, are commonly used to detect publication bias by intuitive examination for asymmetry. However, one of the criticisms of the funnel plot method is that it is subjective and that the same graph may be interpreted differently by different observers.^4^ Alternatively, statistical methods such as Begg’s, Egger’s, and Macaskill’s methods have been proposed to detect publication bias.^3^^,^^5^^,^^6^ However, little is known about the statistical power and thresholds of these tests for detecting such bias. Preceding studies have been using a cut-off p-value of 0.1 as the positive threshold of bias to assess the power of statistical tests, but this value was arbitrarily chosen and has not been systematically validated.^7^ Moreover, there is no consensus about which test is better at detecting publication bias.

The present study aims to evaluate the statistical and discriminatory powers of Begg’s, Egger’s, and Macaskill’s methods, three statistical tests used to detect publication bias in meta-analyses, by varying cut-off p-vales.

## METHODS

To evaluate the test performance of Begg’s, Egger’s, and Macaskill’s methods, visual interpretation of funnel plots was used as a reference standard. A funnel plot is a graphical presentation of each trial’s effect size against one of the sample size measures, such as the precision of the effect size estimate, the overall sample size, and the standard error.^8^ It is based on the assumption that the results from smaller studies will be more widely spread around the mean effect because of a large random error. If there is no publication bias, a plot of sample size versus treatment effect from individual studies in a meta-analysis should therefore look like an inverted funnel.^9^ In practice, smaller studies or non-significant studies are less likely to be published and data for the lower left-hand corner of the graph are often lacking, creating an asymmetry in the funnel shape. It has been reported that different observers may interpret funnel plots differently;^4^ however, we aimed to resolve this problem by having different observers judge the shape of funnel plots and reach a consensus.

All completed systematic reviews that were contained in the Cochrane Database of Systematic Reviews 2002 issue were examined.^10^ Only reviews including 10 or more trials with a binary outcome measure were included in the assessment. The cut-off of 10 trials is based on the minimum number reported by Sutton, who evaluated the effect of publication bias on the results and conclusions of systematic reviews and meta-analyses.^8^ At most, a single meta-analysis from each systematic review was included, and when more than one meta-analysis met the inclusion criteria, the one containing the largest number of studies was selected. If two or more analyses contained the same number of studies, the one with the strongest relation to the primary outcome of the study was selected. One evaluator extracted data from each selected meta-analysis. The binary data of the meta-analysis were used for the analysis, and a continuity correction of 0.5 was used when necessary.^11^

For each review article selected, funnel plots were constructed by plotting the effect measure (eg, the natural logarithm of the odds ratio) against the inverse of its standard error, which is less likely to give a biased result than the use of other effect measures (e.g., log risk ratio and risk difference).^12^ Two observers, one of the authors and another person blinded to the results of statistical analysis, interpreted all funnel plots and judged independently whether publication bias was present. Both observers are general internists and have the experience of conducting meta-analyses that have been published in peer-reviewed journals. To verify consistency, observer A interpreted all funnel plots again 7 days later. Inconsistencies between the observers were resolved by discussion.

Some resulting funnel plots were typical in shape and thus easy to evaluate, but others were atypical and difficult to evaluate. To assess the difficulties of interpretation, two observers also ranked the graphs by using three categories of complexity ([1] easy; [2] moderate; and [3] complicated). The rankings by two observers were then totaled and the result was used as a score of the graph’s complexity. For example, when a graph was ranked “[1] easy” by observer A and “[3] complicated” by observer B, the complexity score for that graph was 4. Therefore a larger number implied a higher level of complexity of a graph’s interpretation.

The asymmetry of the funnel plots was statistically evaluated by three test methods: Begg’s, Egger’s, and Macaskill’s. Begg’s method tests publication bias by determining whether there is a significant correlation between the effect size estimates and their variances. The effect estimates were standardized to stabilize the variance, and an adjusted rank correlation test was then performed.^5^^,^^13^ Let ti and _vi_ be the estimated effect sizes and those variances from the k studies in the meta-analysis, i=1,…..,k. To construct a valid rank correlation test, it is necessary to stabilize the variance by standardizing the effect size prior to performing the test. We correlate t_i_* and _vi_,where

$$t^{*}{}_{i}=(t_{i}-\bar{t})/(v^{*}{}_{i}{)}^{1/2}$$

where

$$\bar{t}=\left(\sum v_{j}{}^{-1}t_{j}\right)/\sum v_{j}{}^{-1}$$

and where v_i_* = v_i_ -(*∑*v_j_^-1^)^-1^ is the variance of t_i_-$\bar{t}$.

Throughout, we have used the rank correlation test based on Kendall’s tau. This involves enumerating the number of pairs of studies that are ranked in the same order with respect to the two factors (i.e., t* and v).

Egger’s method detects funnel plot asymmetry by determining whether the intercept deviates significantly from zero in a regression of the standardized effect estimates versus their precision.^14^ The standard normal deviate (SND), defined as the odds ratio divided by its standard error, is regressed against the estimate’s precision, the latter being defined as the inverse of the standard error (regression equation: SND=a+b×precision). The analysis could be weighted or unweighted by the inverse of the variance of the effect estimates. The unweighted model was used for the current analysis.

Macaskill’s method is fitting a regression directly to the data by using the treatment effect (t_i_) as the dependent variable and the study size (n_i_) as the independent variable.^6^ The observations are weighted by the reciprocal of the pooled variance for each study, that is, the variance of the pooled estimates resulting from combining the data for the two groups. When there is no publication bias, the regression slope has an expected value of zero, and a nonzero slope would suggest an association between effect and sample size, possibly because of publication bias.

We compared these three statistical tests in three different ways. First, we used a p-value as a cut-off point for defining the presence of publication bias by Begg’s method, Egger’s method, or Macaskill’s method, and evaluated a trade-off between sensitivity and specificity by varying cut-off p-values (0.05, 0.1, 0.2). Second, we estimated the sensitivities of these tests corresponding to a fixed false positive rate (0.05 or 0.1) to compare their statistical powers. Third, the receiver operating characteristic curve analysis was used to determine the discriminatory power of each test. A receiver operating characteristic analysis is a popular method of assessing the predictive power of a test by plotting the sensitivity (power) of the test against the corresponding false-positive rate (1-specificity) as the cut-off level of the model varies.^15^ In the present analysis, sensitivity refers to the percentage of systematic reviews with publication bias detected by a statistical test using a given cut-off point out of all systematic reviews with publication bias defined by the reference standard. Specificity refers to the percentage of systematic reviews found by a statistical test to be without publication bias out of all systematic reviews without publication bias defined by reference standard. We compared areas under receiver operating characteristic (ROC) curves by setting the Egger’s method as a reference, using an algorithm suggested by DeLong, DeLong, and Clarke-Pearson.^16^ We defined statistical significance to be p<0.05 for this analysis.

In the evaluation of statistical tests, we used a subgroup of graphs that were scored at 2 and with the observers’ agreements, i.e., both observers agreed that these graphs were reliable and easy to evaluate. We also evaluated three tests by using all 130 reviews as sensitivity analyses. We tested the reliability and validity of the reference standard by examining intra- and inter-observer agreement of these plots, using Kappa statistics.^17^ All statistical analyses were performed with STATA^®^, version 7 (STATA corporation, College Station, TX, USA).

## RESULTS

At the time of investigation, the Cochrane Library (2002, Issue 1) contained 1297 completed systematic reviews. Of these, 130 meta-analyses included 10 or more trials with at least 1 dichotomous outcome. Summary characteristics of the systematic reviews were shown in Table 1. In all 130 reviews, a total of 2,468 original studies evaluated 5,490,223 subjects; the median number of subjects per review was 2,801.5 (range, 3821,874,547); the median number of original studies included in one review was 13 (range, 10-135); the median pooled odds ratios was 0.895 (range, 0.09-6.22).

**Table 1.** Summary characteristics of the systematic reviews included in the current analyses.

Summary indices	36 reviews	130 reviews
The number of subjects per review		
Total	2,874,006	5,490,223
Median	4895	2801.5
Range	538-1,874,547	382-1,874,547

The number of original studies per review		
Total	733	2468
Median	14.5	13
Range	10-70	10-135

Pooled Odds Ratio		
Median	0.825	0.895
Range	0.09-6.22	0.09-6.22

Table 2 shows the number of funnel plots judged to show publication bias by inspection of funnel plots of all studies or of studies restricted to the groups of various scores. When all data were included, the number of studies interpreted as biased by observer B was larger than that according to observer A. Of all 130 graphs, 38 (29.2%) were scored two, 57 (43.8%) were scored three, 27 (20.8%) were scored four, and 8 (6.2%) were scored five.

**Table 2.** Interpretations of funnel plots by different observers.

Observer	Interpretation of funnel plots					Total

	Presence of publication bias (%)*			Absence of publication bias (%)*		
			All data			
Observer A-1	75	(57.7)		55	(42.3)	
Observer A-2	64	(49.2)		66	(50.8)	130
Observer B	95	(73.1)		35	(26.9)	

			Score 2			
Observer A-1	30	(23.1)		8	(6.2)	38
Observer B	32	(24.6)		6	(4.6)	

			Score 3			
Observer A-1	30	(23.1)		27	(20.8)	57
Observer B	43	(33.1)		14	(10.8)	

			Score 4			
Observer A-1	12	(9.2)		15	(11.5)	27
Observer B	19	(14.6)		8	(6.2)	

			Score 5			
Observer A-1	3	(2.3)		5	(3.8)	8
Observer B	3	(2.3)		5	(3.8)	

*: Percentages out of all 130 reviews.

A-1: the interpretation by observer A on the day 1.

A-2: the interpretation on the day 2 by observer A.

Table 3 summarizes the intra- and interobserver agreement of the graphical test. The intraobserver agreement rate was 82.3%, and the Kappa value of observer A was and 0.65 (95% confidence interval (CI), 0.52-0.77). The interobserver agreement rate and the Kappa value on all graphs evaluated by observer A and observer B were 73.8% and 0.42 (95% CI. 0.27-0.57), respectively. This increased to 92.1% and 0.75 (0.49-1.00) when limited to the graphs scored as 2. The intraobserver agreements on all graphs and that on graphs scored as 2 were in the upper range of good reproducibility.^12^ Of the 38 graphs scored at two, 36 with observers’ agreement were used for the main analyses.

**Table 3.** Intra- and inter-observer agreement of interpritating funnel plots.

Data set	Observed	Kappa
	Agreement (%)	(95% CI)
Intra-observer agreement (Observer A)		
All data	82.3	0.65 (0.52-0.77)
Score 2	86.8	0.68 (0.43-0.93)

Inter-observer agreement (Observer A vs. B)		
All data	73.8	0.42 (0.27-0.57)
Score 2	92.1	0.75 (0.49-1.00)
Score 2 or 3	81.1	0.56 (0.39-0.73)
Score 2, 3, or 4	74.6	0.44 (0.28-0.60)
Score 3	73.7	0.46 (0.25-0.67)
Score 4	51.9	0.08 (-0.245-0.40)
Score 5	50.0	-0.07 (-0.75-0.62)

CI: confidence interval.

**Figure 1.**
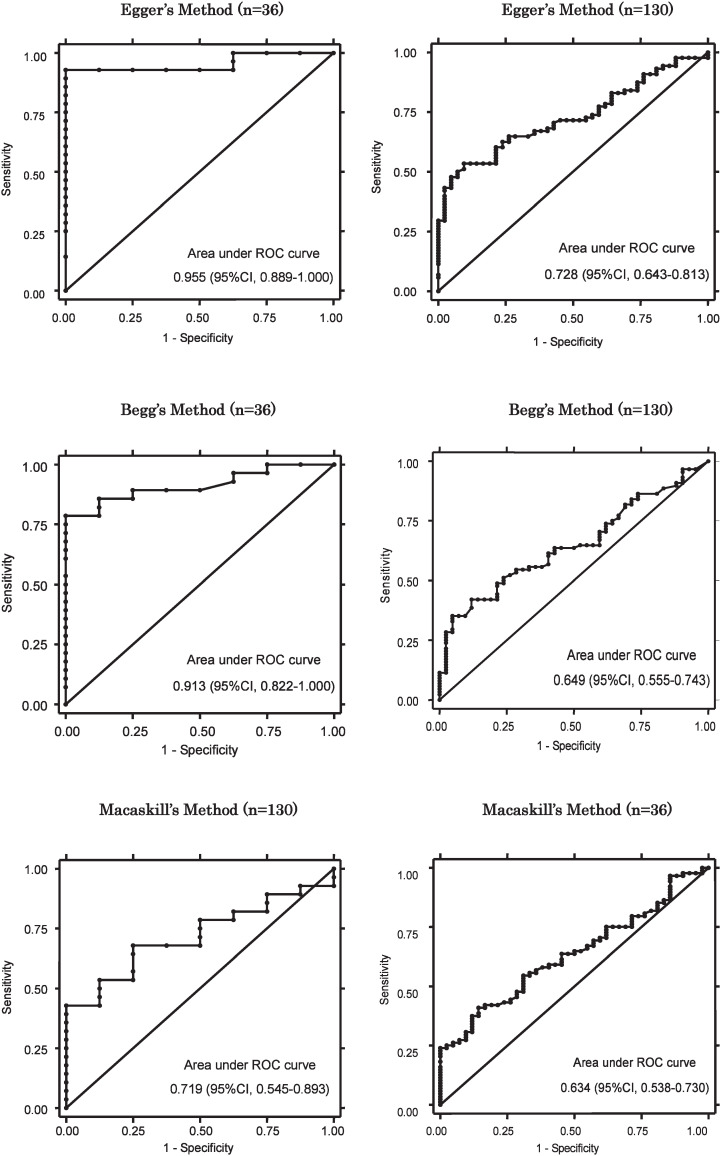
Receiver-operating characteristic (ROC) curves for Begg’s method, Egger’s method and Macaskill’s method. Left column: Main analyses by using funnel plots hat were scored as 2 and with observers’ agreement. Right column: Sensitivity analyses by using all 130 reviews. CI: confidence interval.

As shown in Figure 1, in analyses using data with interobserver agreement (n=36), the area under the ROC curve of Egger’s method was 0.955 (95% CI, 0.889-1.000) and was not different from that of Begg’s method (area=0.913, p=0.2302), but it was larger than that of Macaskill’s method (area=0.719, p=0.0116). In all data set (n=130), the area under the ROC curve of Egger’s method was 0.728 (95% CI, 0.643-0.813), but was not statistically different from that of Begg’s method (area=0.649, p=0.0779) or Macaskill’s method (area=0.634, p=0.0645).

Table 4 summarizes a trade-off between the sensitivities and specificities of three statistical tests by varying the cut-off p-values. All these statistical tests had high specificities with varying degrees of sensitivities. In analyses using data with interobserver agreements (n=36), sensitivities increased as the cut-off p-values increased without a decrement of specificities for any statistical tests. For example, when the cut-off p-value was increased to 0.20 from 0.05, the sensitivity of Egger’s test increased by 0.39, but false positive rate (1-specificity) remained constant. At any cut-off p-values shown in this table, the sensitivity of Egger’s test was larger than that of Begg’s method or Macaskill’s method. These results were not influenced by sensitivity analyses using all data set; false positive rate (1-specificity) was always below the cut-off p-value (the nominal significance level).

**Table 4.** Sensitivities and specificities of statistical tests by varying cut-off points.

Cut-off p-values	Egger		Begg			Macaskill	
		
	Sensitivity	Specificity	Sensitivity		Specificity	Sensitivity	Specificity
			Main analysis (n=36)*				
0.05	0.54	1.00	0.46	1.00		0.21	1.00
0.10	0.71	1.00	0.46	1.00		0.29	1.00
0.15	0.85	1.00	0.64	1.00		0.39	1.00
0.20	0.93	1.00	0.75	1.00		0.43	1.00

			All data set (n=130)				
0.05	0.23	1.00	0.17	0.98		0.08	1.00
0.10	0.38	0.98	0.24	0.98		0.13	1.00
0.15	0.47	0.95	0.27	0.98		0.22	1.00
0.20	0.53	0.90	0.38	0.88		0.25	0.95

* Only reviews that were scored 2 and with inter-observer agreement were used

Table 5 summarizes the sensitivities of statistical tests that the false positive rates (1-specificity) were fixed at 0.05 or 0.1. The statistical power (sensitivity) of Egger’s method was larger than Begg’s or Macaskill’s method, regardless of false positive rates (0.05 or 0.1) or type of data set used (n=130 or n=36).

**Table 5.** Sensitivities of statistical tests given the fixed false positive rates (1-specificity).

1-Specificity (false positive rate)	Sensitivity (corresponding cut-off p-values)		

	Egger	Begg	Macaskill
		Main analysis (n=36)*	
0.05	0.93 (0.20)	0.79 (0.21)	0.43 (0.30)
0.10	0.93 (0.22)	0.86 (0.34)	0.43 (0.31)

		All data set (n=130)	
0.05	0.48 (0.16)	0.35 (0.17)	0.26 (0.20)
0.10	0.53 (0.20)	0.35 (0.20)	0.31 (0.29)

* Only reviews that were scored 2 and with inter-observer agreement were used

**Figure 2.**
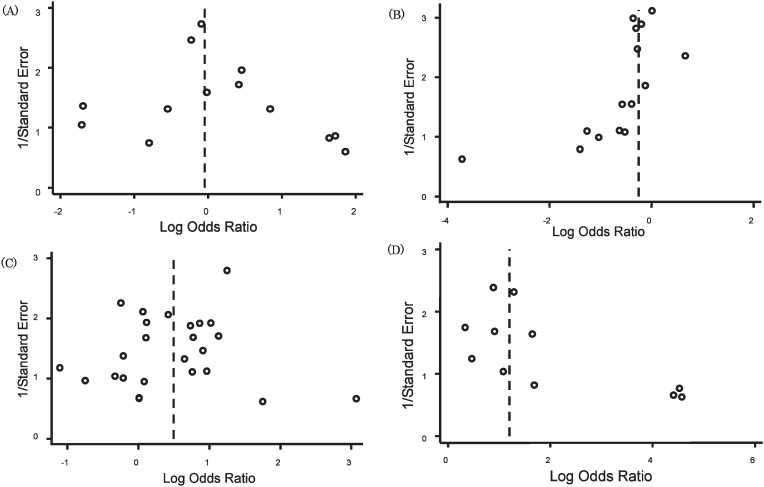
Representative examples of funnel plots in our analysis. (A) A typical example of the absence of publication bias. The number of included studies was 13; the total sample size was 855; the median sample size per review was 46 (range, 20-234); the pooled odds ratio was 0.96 (95% CI, 0.68-1.35). The funnel plots were scored at 4, and both observers agreed that there is no publication bias in this analysis. The p-values were 0.583, 0.641, and 0.603, respectively, for Egger’s method, Begg’s method, and Macaskill’s method. (B) A typical example of the presence of publication bias. The number of included studies was 15; the total sample size was 1278; the median sample size per review was 73 (range, 23-158); the pooled odds ratio was 0.78 (95% CI, 0.61-1.00). The funnel plots were scored at 2, and both observers agreed that there is a publication bias in this analysis. The p-values were respectively 0.006, 0.002, and 0.02 for Egger’s method, Begg’s method, and Macaskill’s method. (C) An example of the inconsistency between two observers in the interpretation of funnel plots. The number of included studies was 25; the total sample size was 2478; the median sample size per review was 97 (range, 36-200); the pooled odds ratio was 1.64 (95% CI, 1.28-2.11). The funnel plots were scored at 3, and observers A asserted that there was publication bias in this analysis, whereas observer B did not. p-values were respectively 0.500, 0.944, and 0.419 for Egger’s method, Begg’s method, and Macaskill’s method. (D) An example of the inconsistency between the three statistical tests in detecting publication bias. The number of included studies was 11; the total sample size was 619; the median sample size per review was 33 (range, 20-204); the pooled odds ratio was 3.32 (95% CI, 2.24-4.92). The funnel plots were scored at 2, and both observers agreed that there is no publication bias in this analysis. The p-values were respectively 0.018, 0.020, and 0.352 for Egger’s method, Begg’s method, and Macaskill’s method. With positivity criterion p<0.1, Egger’s method and Begg’s method suggest the presence of a publication bias, but Macaskill’s test did not.

Figure 2 shows representative examples of funnel plots in the current analyses. For example, graph (D) is an example of the discrepancy between the three statistical tests in terms of detecting publication bias. The number of included studies in this review was 11; the total sample size was 619; the median sample size per review was 33 (range, 20-204); the pooled odds ratio was 3.32 (95% CI, 2.24-4.92). The funnel plots were scored as 2, and both observers agreed that this analysis had publication bias. The p-values were respectively 0.018, 0.020, and 0.352 for Egger’s, Begg’s, and Macaskill’s method. For a cut-off p-vale of 0.1, Egger’s method and Begg’s method suggest the presence of a publication bias, but Macaskill’s method does not.

## DISCUSSION

Publication bias in meta-analysis could lead to serious consequences, and there have been repeated calls for a worldwide registration of clinical trials.^18^^-^^22^ Recently the International Committee of Medical Journal Editors (ICMJE) proposed comprehensive trial registration as a solution to the bias problem. ICMJE member journals will require, as a condition of consideration for publication, registration in a public trials registry.^3^ This policy applies to any clinical trial starting enrolment after July 1, 2005. An examination of possible publication bias and related bias should be an essential part of meta-analyses and systematic reviews. Although a registration of trials and the creation of a database of all published and unpublished trials could solve the problem, it will be a long time before these goals are completely fulfilled. The methodology of assessing publication bias is still developing, and the current study generates the following suggestions concerning how to evaluate publication bias.

First, our results showed that Egger’s method had stronger statistical power than Begg’s method or Macaskill’s method. This is in part consistent with the reports by Egger, Sterne, or Macaskill,^2^^,^^6^^,^^7^ which suggested the difference in power between Egger’s method and Begg’s or Macaskill’s method. In the Macaskill report, however, which has compared these three statistical tests, the use of Macaskill’s method was recommended to detect publication bias because it had a low false positive rate; Begg’s and Egger’s methods had higher false positive rate than the nominal level, though they had stronger statistical powers. Our results, however, showed that the statistical power of Egger’s or Begg’s method seemed stronger than the method given the fixed false positive rates (0.05 or 0.1), which is inconsistent with Macaskill’s opinion that the stronger statistical power of these two tests could be attained by the trade-off with problematically high false positive rate. It is not clear why our results are inconsistent with Macaskill’s report, but a possible reason may be that they evaluated their method by using simulated data, whereas we have used data in a real setting. For example, in their report, most of the simulated reviews included only a few hundred of subjects per study, but the median number of subjects in the current analysis was 2,801.5; our analysis using data in a real setting included a broader range of effect size (OR: 0.09-6.22) than that in their simulation study (OR: 0.25-1.0). Our results thus may suggest that their method is not necessarily preferred in a real setting. The results of the area under the receiver operating characteristics curve analyses have also revealed that Begg’s or Egger’s method had stronger discriminatory power in detecting publication bias than Macaskill’s method did.

Second, we have shown that a higher statistical power could be attained with a relatively small increment of false positive rate (type I error) by setting cut-off p-values of three statistical tests larger than conventionally used cut-off p-value. For example, by increasing it to 0.15 from 0.05, the sensitivity of Egger’s test increased by 0.24, but the false positive rate increased by only 0.05 in all data set. The same happens with Begg’s and Macaskill’s method. Typically, the cut-off p-value of 0.1 has been used to determine the presence of publication bias by the use of these statistical tests,^23^ the conventional cut-off point of these statistical tests could be reconsidered depending on the purpose of using them.

It is important to determine a reference standard for evaluating sensitivities and specificities when evaluating test performance. Funnel plots, the simplest and most commonly used method in meta-analysis, were used as a reference standard in the current analysis for the following reasons. First, under certain conditions, they have higher reproducibility than generally thought; this observation is based on multiple observer conclusions.^4^ Observer agreements on the interpretation of funnel plots have not been systematically evaluated prior to this study. Our inter-observer agreement rate was and 92.1%, and Kappa value of the graphs scored 2 was and 0.75. Although observer agreement decreased with the degree of difficulty of interpretation (the easier the graphs were to interpret, the better the reproducibility of the funnel plots), it was still 73.8% even when all 130 meta-analyses were used; the Kappa value was 0.42, which denotes good reproducibility. This justifies our use of funnel plots as a reference standard.

Second, funnel plots are suitable for determining the presence or absence of publication bias because they are more than a tool for evaluating an asymmetry, which statistical tests mainly evaluate. Publication bias has long been considered to exist when funnel plots are asymmetrical.^9^ However, many factors influence their asymmetry. For example, English-language bias – the preferential publication of negative findings in journals published in languages other than English – makes the location and inclusion of non-English studies in meta-analysis less likely.^24^ Also as a consequence of citation bias, negative studies tend to be quoted less frequently and are therefore more likely to be missed in a literature search.^25^^,^^26^ Other factors causing an asymmetry of funnel plots include poor methodological design of small studies, inadequate analysis, fraud, or choice of effect measure. Therefore to relate the asymmetry to publication bias, other factors should be evaluated; for that purpose, a funnel plot does a better job than a statistical test because the observer can judge publication bias subjectively by referring to this information.

There are several limitations in this study. First, we did not include meta-analyses involving a small number of studies, since the power of statistical tests declines when only a few studies are subjected to meta-analysis,^7^ and graphical interpretation may be difficult and biased when the number of studies is small. Different results of test performance may arise if these few studies are included. Second, it is an issue that the larger a cut-off p-value, the higher the probability of detecting publication bias by chance, but our results suggested that the benefits of using a larger cut-off p-value surpass the problems of false positivity for publication bias. Further evaluation is necessary to verify whether our results can be extrapolated to funnel plots other than those used in our analyses.

In conclusion, Egger’s linear regression method or Begg’s method had higher statistical and discriminatory power for detecting publication bias than Macaskill’s method given the same type I error level. The false negative rate of these methods could be improved by increasing the cut-off p-value without a substantial increment of false positive rate.
